# An algorithm as a diagnostic tool for central ocular motor disorders, also to diagnose rare disorders

**DOI:** 10.1186/s13023-019-1164-8

**Published:** 2019-08-08

**Authors:** Ludwig Kraus, Olympia Kremmyda, Tatiana Bremova-Ertl, Sebastià Barceló, Katharina Feil, Michael Strupp

**Affiliations:** 1 Department of Neurology and German Center for Vertigo and Balance Disorders, Ludwig-Maximilians University, Munich, Campus Grosshadern, Marchioninistr. 15, 81377 Munich, Germany; 2 Department of Neurology, Inselspital, University Hospital Bern, Bern, Switzerland; 3 Syntax for Science, Parc Bit, Edif. Disset A2, 07121 Palma de Mallorca, Spain

**Keywords:** Ocular motor disorder, Algorithm, Niemann pick type C, Gaucher’s disease type 3, Ataxia teleangiectasia, Ataxia with oculomotor apraxia, Progressive supranuclear palsy, Wernicke encephalopathy

## Abstract

**Background:**

Recently an increasing number of digital tools to aid clinical work have been published. This study’s aim was to create an algorithm which can assist physicians as a “digital expert” with the differential diagnosis of central ocular motor disorders, in particular in rare diseases.

**Results:**

The algorithm’s input consists of a maximum of 60 neurological and oculomotor signs and symptoms. The output is a list of the most probable diagnoses out of 14 alternatives and the most likely topographical anatomical localizations out of eight alternatives. Positive points are given for disease-associated symptoms, negative points for symptoms unlikely to occur with a disease. The accuracy of the algorithm was evaluated using the two diagnoses and two brain zones with the highest scores. In a first step, a dataset of 102 patients (56 males, 48.0 ± 22 yrs) with various central ocular motor disorders and underlying diseases, with a particular focus on rare diseases, was used as the basis for developing the algorithm iteratively. In a second step, the algorithm was validated with a dataset of 104 patients (59 males, 46.0 ± 23 yrs). For 12/14 diseases, the algorithm showed a sensitivity of between 80 and 100% and the specificity of 9/14 diseases was between 82 and 95% (e.g., 100% sensitivity and 75.5% specificity for Niemann Pick type C, and 80% specificity and 91.5% sensitivity for Gaucher’s disease). In terms of a topographic anatomical diagnosis, the sensitivity was between 77 and 100% for 4/8 brain zones, and the specificity of 5/8 zones ranged between 79 and 99%.

**Conclusion:**

This algorithm using our knowledge of the functional anatomy of the ocular motor system and possible underlying diseases is a useful tool, in particular for the diagnosis of rare diseases associated with typical central ocular motor disorders, which are often overlooked.

**Electronic supplementary material:**

The online version of this article (10.1186/s13023-019-1164-8) contains supplementary material, which is available to authorized users.

## Background

Clinical practice shows that the diagnosis of rare diseases and central ocular motor disorders is often difficult, even for neurologists. On the other hand, we do have detailed knowledge on the anatomy, physiology and pathophysiology of ocular motor disorders, which allows a precise topographic anatomical diagnosis based on bedside examination even without any laboratory examinations [1] (see Table 3 for a short description of the most important parts of the clinical oculomotor examination). This means that, on the basis of clinical information, we can determine whether there is an impairment in the midbrain, pons, medulla or the cerebellar flocculus, nodulus, vermis, or fastigial nucleus.

Rare diseases, such as Niemann-Pick type C (NPC) [2], Tay-Sachs (TS) or Gaucher’s disease type 3 (GD 3), are often overlooked, although the diagnosis can often be made on the basis of the patient history and clinical examination and confirmed by genetic testing. Several of these diseases are characterized by quite specific ocular motor findings, such as a supranuclear saccade or – at a later stage of the disease – gaze palsy in NPC and TS (for reference see [1]). From a therapeutic point of view, these diseases should also not be overlooked because several of them are treatable nowadays [3, 4].

Facing these problems, we designed a simple and easy-to-use algorithm to help clinicians to correctly diagnose central ocular motor disorders and, in particular, associated rare diseases. Similar approaches have been recently used to diagnose cerebellar ataxias [5] or vertigo and dizziness [6].

## Methods

The algorithm was created in three steps.

### Step one

Two lists were designed: list A contained 14 diseases which often present with ocular motor disorders, list B contained 60 signs and symptoms typically found in these diseases. The latter can be subdivided into two major groups: general and ocular motor signs and symptoms (see Additional file 1).

Subsequently a table with list A in the cross column and list B in the along column was developed. Based on the current literature [1, 7, 8], we linked the symptoms to the diseases by simply entering “Yes” if the symptom occurs with the disease and “No” if it does not.

By including various diseases, we wanted to give a representative clinical overview. Of course, the onset varies greatly depending on the etiology. In terms of imaging, even MRI of the brainstem might be normal within the first 72 h after symptom onset [9], which makes a systematic clinical examination and topographic diagnosis even more relevant. We included the following 14 different diseases: Niemann-Pick disease type C (NPC) [10, 11], ataxia teleangiectasia (AT) [12–14], ataxia with oculomotor apraxia 1 and 2 (AOA 1,2) [15], Gaucher’s disease type 3 (GD 3) [16, 17], Tay-Sachs disease (TS) [18], Wernicke encephalopathy [19, 20], Huntington’s chorea [21], multiple sclerosis (MS) [22, 23], Parkinsonian syndromes [24], progressive supranuclear palsy (PSP) [25], tumor, infarction/hemorrhage, inflammatory encephalitis and various cerebellar syndromes (the latter term sums up diseases which are not a single point on our list A but cause a cerebellar syndrome like the spinocerebellar ataxias, CANVAS (Cerebellar ataxia with neuropathy and vestibular areflexia [26]) and Chiari malformation).

The basic working principle of the algorithm was to create a score for all of the 14 diseases as an output following the input of a patient’s signs and symptoms. The symptoms are entered into an entry mask with “Yes” if the patient suffers from a symptom, “No” if he does not and “0” if a symptom was not tested or not testable (see Additional file 3).

The algorithm was further improved by increasing the strength of the linking of very typical symptoms to certain diseases. In the above-mentioned table we entered not “Yes” but “HR” for “highly related”. If this symptom occurred, two points instead of one were added to a disease’s score. We implemented this linking with, e.g., “internuclear ophthalmoplegia, aged < 60 years” and “MS”, “vertical saccade palsy” and “NPC”, “resting tremor” and “Parkinsonian syndromes”. We also implemented a negative linking meaning that if a certain symptom occurs, the score of a disease was decreased. If, for instance, “paresis” occurs, the score of “NPC” and “GD3” is decreased by two points to better differentiate it from “TS”.

### Step two

The first version of the algorithm was improved using the data from 102 patients (56 males, 48.0 ± 22 yrs., distribution of the diseases: NPC - 7, AT - 5, AOA1,2–5, GD3–7, TS - 5, Wernicke encephalopathy - 5, Huntington’s chorea - 6, MS - 10, Parkinson syndromes - 9, PSP - 9, tumor - 4, infarction/hemorrhage - 9, inflammatory encephalitis - 5, various cerebellar syndromes - 16). Most of these patients had been examined at our University Hospital in the past, independently of this study [3, 17]. We went through the documented oculomotor examinations and looked for patients who fulfilled our criteria. There were two inclusion criteria: 1. they had to be diagnosed with one and only one of the diseases in list A, and 2. they had to have oculomotor disorders which were found and described exactly in the documentation of the examination. The following exclusion criterion applied: patients had not to have had a second condition causing oculomotor disorder, such as brain surgery or a stroke in the past.

We put the clinical findings from these patients into the entry mask of the algorithm and evaluated its output. Then we adjusted the algorithm in an iterative way until we reached a good sensitivity and specificity. The arithmetic procedures we used in the algorithm were adding zero, one, two, three or four points to the score or subtracting one, two or three points.

### Step three

This was a repetition of step two without further adjustment of the algorithm. We tested if similar results could be reproduced with a second cohort of 104 patients (59 males, 46.0 ± 23 yrs., distribution of the diseases: NPC - 10, AT - 5, AOA1,2–4, GD3–10, TS - 5, Wernicke encephalopathy - 5, Huntington’s chorea - 5, MS - 10, Parkinson syndromes - 10, PSP - 10, tumor - 4, infarction/hemorrhage - 11, inflammatory encephalitis - 5, various cerebellar syndromes - 10).

We used the same approach as described above to make the algorithm produce a suggestion on the topographical anatomical localization of the lesion. List B with the symptoms remained exactly the same, while list A with the diseases was changed into a list of brain zones, which, when affected, result in ocular motor disorders. Again we used current literature to link the symptoms to the eight zones: midbrain, pons, medulla oblongata, basal ganglia, frontoparietal cortex and the three parts of the cerebellum flocculus/paraflocculus, vermis/fastigial nucleus and nodulus/uvula [8] (see Additional file 2).

We postulated three rules for interpreting the algorithm’s result for the diseases: 1. The result consists of the two diseases which get the highest scores in the output list (see Additional file 4). This can be more than two diseases if several get the same score. 2. If the algorithm provides more than five diseases as the result, we considered this as not helpful. When calculating the diseases’ sensitivity and specificity we counted such results as false negatives for the actual disease and as false positives for the other 13 diseases. 3. If one disease’s score was at least three points higher than any other score, this disease was considered as the only result of the algorithm. When the correct diagnosis appeared in the above-defined result of the algorithm consisting of one to five diseases we counted the result as a true positive for the actual disease and a true negative for the other diseases that did not appear in the result. Every incorrect one of the one to five result-diseases was counted as a false positive.

To interpret the algorithm’s result for the topographic anatomical location we also postulated three rules similar to but not identical to the rules for the disease: 1. The result consists of the two brain zones which get the highest scores in the algorithm’s output list. This can be more than two zones if several get the same score. 2. Every score with only one point or less is ignored unless one point is the highest existing score. 3. If the algorithm provides more than four zones as a result, we considered this as not helpful and treated it as mentioned above. The sensitivity and specificity were calculated in the same way as for the diseases described above.

Approval from the ethics committee board of the University of Munich was obtained for the study. All investigations were conducted according to the principles of the Declaration of Helsinki.

### Statistical analysis

**Fig. 1:**
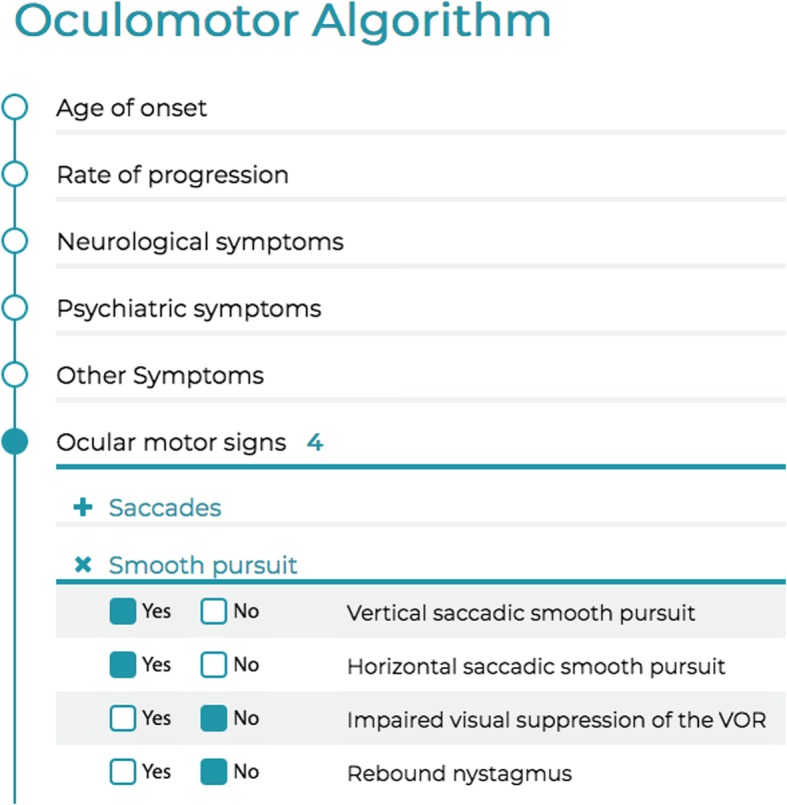
Screenshot of the data entry mask in the finished web tool. This excerpt shows the main signs and symptoms categories of the data entry file. By clicking on “Yes” or “No” one confirms or denies a symptom. Symptoms that were not tested can just be skipped by not clicking on any of the possibilities and leaving the field empty

For the statistical evaluation, the software “SAS” v9.3 was used. We calculated the confidence limits of the sensitivity/specificity using an asymptotic normal approximation to the binomial distribution. The whole algorithm was then embedded in an easy-to-use web tool which can be seen in Fig. 1 (called ADOC – *A*lgorithm for the *D*iagnosis of *OC*ulomotor disorders).

## Results

As mentioned in Methods, the algorithm to diagnose the affected brain structures and diseases was developed in an iterative way. In the following, the sensitivity and specificity are given for the last version.

### First (“testing”) cohort

In the testing cohort, the sensitivity for the brain zones lay between 90 and 0% (best: frontoparietal cortex 90%, basal ganglia 80%, flocculus/paraflocculus 75%). The specificity was between 98 and 49% (frontoparietal cortex and nodulus/uvula 98%, basal ganglia 96%; and pons 49%).

For the diseases, the sensitivity ranged from 100% (NPC, AT, AOA1 and 2, GD 3, TS, PSP, Wernicke’s encephalopathy, inflammatory encephalitis, infarction /hemorrhage) to 75% (tumor).

As our result design consists of at least two suggestions about the underlying disease in most cases, there was at least one false positive in every output. So, as expected, the specificity was not as high, ranging from 96 to 63% (best: infarction/hemorrhage and Parkinsonian syndromes 96%, Wernicke’s encephalopathy 95%; and MS 63%).

### Second (“validation”) cohort

The sensitivity and specificity of diseases and brain zones from the validation cohort can be seen in Tables 1 and 2 respectively. For the brain zones, the sensitivity ranged from 100 to 0% (medulla oblongata 100%, pons 82%, basal ganglia 79%). The specificity was between 99 and 52% (frontoparietal cortex 99%, nodulus/ uvula 98%, medulla oblongata 84%; and midbrain 52%).

**Table 1:** Sensitivity and specificity for the diseases in the validation cohort. Sensitivity ranged from 100% for NPC, AOA1 and 2, TS, Wernicke’s encephalopathy, inflammatory encephalitis, infarction/hemorrhage to 60% for AT. Specificity was between 95% for Parkinsonian syndromes and Huntington’s chorea and 66% for inflammatory encephalitis. Additionally the 95% confidence interval was calculated for every value

	Niemann-Pick disease Type C (NP-C)	Inflammatory Encephalitis	Tumor	Infarction/ hemorrhage	Multiple sclerosis	Parkinsonian syndromes	Progressive supranuclear palsy (PSP)
Sensitivity:	10/10	5/5	3/4	11/11	8/10	8/10	8/10
	100%	100%	75.0%	100%	80.0%	80.0%	80.0%
95% CI	1.000–1.000	1.000–1.000	0.326–1.000	1.000–1.000	0.552–1.000	0.552–1.000	0.552–1.000
Specificity:	71/94	65/99	82/100	82/93	63/94	89/94	83/94
	75.5%	65.7%	82.0%	88.2%	67.0%	94.7%	88.3%
95% CI	0.668–0.842	0.563–0.750	0.745–0.895	0.816–0.947	0.575–0.765	0.901–0.992	0.818–0.948
	Wernicke’s encephalo-pathy	Ataxia teleangiectasia	Ataxia with oculomotor apraxia 1/2	Gaucher’s disease Type 3 (GD3)	Huntington’s chorea (HTT)	Cerebellar syndromes	Tay-Sachs disease
Sensitivity:	5/5	3/5	4/4	8/10	4/5	10/10	5/5
	100%	60.0%	100%	80.0%	80.0%	100%	100%
95% CI	1.000–1.000	0.171–1.000	1.000–1.000	0.552–1.000	0.449–1.000	1.000–1.000	1.000–1.000
Specificity:	89/99	86/99	78/100	86/94	94/99	79/94	78/99
	89.9%	86.9%	78.0%	91.5%	94.5%	84.0%	78.8%
95% CI	0.840–0.958	0.802–0.935	0.699–0.861	0.858–0.971	0.906–0.993	0.766–0.914	0.707–0.868

**Table 2:** Sensitivity and specificity for the brain zones in the validation cohort. Sensitivity ranged from 100% for medulla oblongata to 0% for nodulus/uvula. Specificity was between 99% for frontoparietal cortex and 52% for midbrain. Additionally the 95% confidence interval was calculated for every value

	Midbrain	Pons	Medulla oblongata	Flocculus/ Paraflocculus	Vermis/ Fastigial Nucleus	Nodulus/ Uvula	Basal Ganglia	Fronto- parietal Cortex
Sensitivity:	30/39	28/34	4/4	34/54	14/54	0/54	22/28	5/11
	76.9%	82.4%	100%	63.0%	25.9%	0.0%	78.6%	45.5%
95% CI	0.637–0.901	0.695–0.952	1.000–1.000	0.501–0.758	0.142–0.376	0.000–0.000	0.634–0.938	0.160–0.749
Specificity:	30/58	34/63	78/93	25/43	34/43	42/43	57/69	85/86
	51.7%	54.0%	83.9%	58.1%	79.1%	97.7%	82.6%	98.8%
95% CI	0.389–0.646	0.417–0.663	0.764–0.913	0.434–0.729	0.669–0.912	0.932–1.000	0.737–0.916	0.966–1.000

For the diseases, the sensitivity ranged from 100 (NPC, AOA1 and 2, TS, Wernicke’s encephalopathy, inflammatory encephalitis, infarction/hemorrhage) to 60% (AT). The specificity was between 95 and 66% (Parkinsonian syndromes and Huntington’s chorea 95%, GD 3 92%; and inflammatory encephalitis 66%). In general, the results of the validation cohort were slightly worse than in the testing cohort, with the biggest difference being the sensitivity for AT (5/5 vs. 3/5) (Table 3).

**Table 3:** Different aspects of the clinical oculomotor examination. This table contains a short description of the most important parts of the clinical oculomotor examination and the possible pathologies which should be looked for

Type of examination	Question
Inspection	
Head/body posture	Tilt or turn of head/body
Position of eyelids	Ptosis
Eye position/motility	
Position of eyes during straight-ahead gaze	Misalignment in primary position, spontaneous or fixation nystagmus
	Horizontal or vertical misalignment
Cover/Uncover test	
Examination of eyes in eight positions (binocular and monocular)	Determination of range of motility, gaze-evoked nystagmus (GEN), end-point nystagmus, sustained, unsustained
Gaze-holding function	
10–40° in the horizontal	GEN: horizontal, also important for the diagnosis of downbeat nystagmus
10–20° in the vertical	vertical
Back to 0° after 30 s	rebound nystagmus
Slow smooth pursuit movements	
Horizontal and vertical	Smooth or saccadic
Saccades	
Horizontal and vertical when looking around or at targets; important to note: upper eye must be lifted when examining vertical saccades	Latency, velocity, accuracy, conjugacy
Optokinetic nystagmus (OKN)	
Horizontal and vertical with OKN drum or tape	Inducible, direction, phase (reversal or monocularly diagonal)
Peripheral vestibular function	
Head-impulse test (HIT) for the examination of the VOR (Halmagyi–Curthoys test): rapid turning of the head and fixation of a stationary target; nowadays better to be done by the video-HIT	Unilateral or bilateral peripheral vestibular deficit
Fixation suppression of the VOR	
Turning the head and fixation of a target moving at same speed	Impairment of fixation suppression of the VOR
Examination with Frenzel’s or the M-glasses [27]	
Straight-ahead gaze, to the right, to the left, downward and upward	Peripheral vestibular spontaneous nystagmus versus central fixation nystagmus
Head-shaking test	Head-shaking nystagmus

## Discussion

The major findings of this study are as follows:

- First, this algorithm can be a helpful tool for diagnosing, in particular, rare diseases associated with central ocular motor disorders. For example, in the validation cohort we reached a sensitivity of 100% for NPC (10/10) and Wernicke’s encephalopathy (5/5). It is assumed that both of them are vastly underdiagnosed [11, 19]. Since these diseases are treatable or, in the case of Wernicke’s encephalopathy, even curable, an early diagnosis has a huge impact on the outcome of these patients.
- Second, the results for the brain zones were generally worse but can still give an indication of where to look for pathologies in imaging. In the validation cohort, the sensitivity for involvement of the medulla oblongata was 100% (4/4) and for the pons 82.4% (28/34).
- Third, the algorithm can be applied in less than 5 min.

Compared to “medx” [6], a similar tool recently published to diagnose vertigo and dizziness, our algorithm showed a higher sensitivity (medx: 40 to 80.5%) but a lower specificity (medx: at least 80%). This can perhaps be explained by the fact that “medx” focuses on the first suggested diagnosis, whereas our tool presents the two top-scoring results. Since our algorithm deals with more rare diseases, the different approaches seem to be suitable for the different problems they are supposed to solve. Another recent algorithm to diagnose recessive ataxias is called “RADIAL” [5]. It showed a higher average sensitivity and specificity (RADIAL: 92.2 and 95.4%, respectively) than our tool but it works with around twice as many features (120 vs. 60).

This study has several limitations: First, it was a retrospective analysis. Second, our gold standard was the diagnosis made in the hospital, which is not flawless. Third, a major problem was that the affected brain zones could not always be verified in the brain imaging available or that patients had multiple lesions as in MS. Regarding the cerebellum, imaging often shows no pathologies, however the clinical signs are often specific based on current knowledge of the function and dysfunction of the flocculus/paraflocculus, nodulus, nucleus fastigii and dorsal vermis. All in all, however, the major focus was on the diagnosis of rare diseases which can evidently be improved by such a simple algorithm.

## Conclusions

In summary, this algorithm uses our knowledge on the functional anatomy of the ocular motor system. It is based on the simple idea of comparing signs and symptoms typical of certain diseases and brain lesions to signs and symptoms occurring in a certain patient. It is a useful tool for diagnosing diseases, in particular rare ones, which present with central ocular motor disorders.

## Additional files

Additional file 1: Workflow of the algorithm – diseases. These tables show the working principle of the algorithm using the example of 5 of the 14 diseases (MS, PSP, Wernicke’s encephalopathy, AT, NPC). Whenever a symptom occurs in a patient whether the disease’s score is increased or decreased depends on the type of linking: NEV = − 3, OTTD = − 2, UL = − 1, N = + 0, Y = + 1, HR = + 2. (DOCX 28 kb)

Additional file 2: Workflow of the algorithm – brain zones. These tables show the working principle of the algorithm using the example of 4 of the 8 brain zones (midbrain, pons, medulla, flocculus/paraflocculus). Whenever a symptom occurs in a patient, whether the brain zone’s score is increased depends on the type of linking: N = + 0, R = + 1, HR = + 2. (DOCX 20 kb)

Additional file 3: Data entry mask with 3 examples. These tables show three sample patients’ signs and symptoms entered in the algorithm’s entry mask. “Yes” means the symptom was present, “No” means it was not and “0″ it was not looked for in the examination. The real diagnoses are:1 = PSP, 2 = Wernicke’s Encephalopathy, 3 = NPC. (DOCX 24 kb)

Additional file 4: Output of the algorithm for the input of the 3 examples from Additional file 3. According to our result interpretation rules (see Methods), the algorithm’s diagnosis suggestions for the three patients are: Patient 1: brain zone: Basal ganglia and pons; disease: PSP. Patient 2: brain zone: Pons, medulla oblongata, flocculus/paraflocculus and vermis/fastigial nucleus; disease: Wernicke’s encephalopathy, MS and inflammatory encephalitis. Patient 3: brain zone: Midbrain, basal ganglia; disease: NPC. The real diagnoses are: 1 = PSP, 2 = Wernicke’s Encephalopathy, 3 = NPC. (DOCX 16 kb)

## Data Availability

All data are available from the corresponding authors on request.

## Abbreviations

ADOC: Algorithm for the diagnosis of oculomotor disorders
AOA 1,2: Ataxia with oculor motor apraxia type 1 and 2
AT: Ataxia teleangiectasia
CANVAS: Cerebellar ataxia with neuropathy and vestibular areflexia
GD3: Gaucher’s disease type 3
MS: Multiple sclerosis
NPC: Niemann-Pick disease type C
PSP: Progressive supranuclear palsy
TS: Tay-Sachs disease
