# Supplementary material for: An algorithm as a diagnostic tool for central ocular motor disorders, also to diagnose rare disorders
Source: Orphanet J Rare Dis. 2019 Aug 8;14:193. doi: 10.1186/s13023-019-1164-8 (PMC6688379; doi:10.1186/s13023-019-1164-8)
Supplement: Supplementary file 1 — Workflow of the algorithm – diseases. These tables show the working principle of the algorithm using the example of 5 of the 14 diseases (MS, PSP, Wernicke’s encephalopathy, AT, NPC). Whenever a symptom occurs in a patient whether the disease’s score is increased or decreased depends on the type of linking: NEV = − 3, OTTD = − 2, UL = − 1, N = + 0, Y = + 1, HR = + 2. (DOCX 28 kb) [file 13023_2019_1164_MOESM1_ESM.docx]

| **Additional File 1.1.**   \|  \| \| --- \| \| | | | **Multiple sclerosis** | **Progressive supra - nuclear palsy (PSP)** | **Wernicke encephalo-pathy** | **Ataxia tele-angiectasia**  **(AT)** | **Niemann-Pick disease  Type C (NPC)** |
| --- | --- | --- | --- | --- | --- | --- | --- | --- |
|  |  |  |  |  |  |  |  |
| **Age of onset** | | Age of onset: <10 years | UL | NEV | UL | Y | Y |
|  |  | Age of onset: ≥10 years | Y | Y | Y | NEV | Y |
| ***Rate of progression*** | | Years to decades | Y | Y | NEV | Y | Y |
|  |  | Months | Y | Y | NEV | Y | Y |
|  |  | Weeks | Y | N | Y | N | N |
|  |  | Days | Y | NEV | Y | N | N |
|  |  | Minutes to hours | Y | NEV | Y | NEV | NEV |
| ***Neurological  symptoms*** | **Extra-  pyramidal symptoms** | Rigidity | N | HR | N | N | N |
|  |  | Spasticity | Y | N | N | N | Y |
|  |  | Akinesia | N | HR | N | Y | Y |
|  |  | Dystonia | N | N | N | Y | Y |
|  |  | Choreatoform | Y | N | OTTD | Y | Y |
|  |  | Resting tremor | Y | UL | N | Y | N |
|  |  | Intention tremor | Y | N | Y | Y | Y |
|  | **Other  neurological symptoms** | Dysarthrophonia/dysphagia | Y | Y | N | Y | Y |
|  |  | Aphasia | Y | N | N | N | N |
|  |  | Paresis (arms/legs/face) | Y | N | N | N | OTTD |
|  |  | Peripheral neuropathy | N | N | N | Y | N |
|  |  | Ataxia | Y | N | Y | HR | Y |
|  |  | Seizures | Y | N | Y | N | Y |
|  |  | Disoriented to time, place,  person or situation | Y | N | HR | N | N |

| **Additional File 1.2.**   \|  \| \| --- \| \| | | | **Multiple sclerosis** | **Progressive supra - nuclear palsy (PSP)** | **Wernicke encephalo-pathy** | **Ataxia tele-angiectasia**  **(AT)** | **Niemann-Pick disease  Type C (NPC)** |
| --- | --- | --- | --- | --- | --- | --- | --- | --- |
|  |  |  |  |  |  |  |  |
| ***Psychiatric  symptoms*** | **Childhood** | Mental retardation  (mild to moderate: IQ 35-69) | N | N | N | N | Y |
|  |  | Mental retardation  (severe to profound: IQ up to 34) | N | N | N | N | Y |
|  |  | Developmental disorder of  speech and language | N | N | N | Y | Y |
|  |  | Developmental disorder of  scholastic skills | N | N | N | Y | Y |
|  |  | Hyperkinetic disorder | N | N | N | N | UN |
|  |  | Conduct disorder  (beginning in childhood) | N | N | N | UN | Y |
|  | **Adulthood** | Amnesic disorder | Y | Y | HR | N | N |
|  |  | Mood disorder | Y | Y | N | N | Y |
|  |  | Psychotic disorder | Y | N | Y | N | Y |
|  |  | Conduct (behavioral) disorder | Y | N | N | UN | Y |
| **Other Symptoms** | | Fever | N | N | N | N | N |

| **Additional File 1.3.**   \|  \| \| --- \| \| | | | **Multiple sclerosis** | **Progressive supra - nuclear palsy (PSP)** | **Wernicke encephalo-pathy** | **Ataxia tele-angiectasia**  **(AT)** | **Niemann-Pick disease  Type C (NPC)** |
| --- | --- | --- | --- | --- | --- | --- | --- | --- |
|  |  |  |  |  |  |  |  |
| **Ocular  motor signs** | **Saccades** | Impaired saccade initiation with  increased latency of saccades  ("ocular motor apraxia") | R | N | N | R | R |
|  |  | Internuclear opthalmoplegia (INO),  aged < 60 years | HR | N | R | N | N |
|  |  | Internuclear opthalmoplegia (INO),  aged >= 60 years | R | N | R | N | N |
|  |  | Horizontal saccade palsy | R | R | R | R | R |
|  |  | Vertical saccade palsy | R | HR | R | R | HR |
|  |  | NO Vertical saccade palsy | N | OTTD | N | N | N |
|  |  | Hypermetric saccades | R | N | N | N | N |
|  | **Smooth  pursuit** | Vertical saccadic smooth pursuit | R | R | R | R | R |
|  |  | Horizontal saccadic smooth pursuit | R | R | R | R | R |
|  |  | Impaired visual suppression  of the vestibulo-ocular reflex (VOR) | R | N | N | UN | N |
|  | **Vestibular  signs** | Pathological Head Impulse  Test (VOR-test) | R | N | R | HR | N |
|  |  | Skew deviation | R | N | N | R | N |
|  |  | Head tilt | R | N | N | UN | N |
|  | **OKN** | Horizontally reduced  optokinetic nystagmus | R | N | R | R | R |
|  |  | Vertically reduced  optokinetic nystagmus | R | HR | R | R | HR |

| **Additional File 1.4.**   \|  \| \| --- \| \| | | | **Multiple sclerosis** | **Progressive supra - nuclear palsy (PSP)** | **Wernicke encephalo-pathy** | **Ataxia tele-angiectasia**  **(AT)** | **Niemann-Pick disease  Type C (NPC)** |
| --- | --- | --- | --- | --- | --- | --- | --- | --- |
|  |  |  |  |  |  |  |  |
| **Ocular  motor signs** | **Gaze  holding** | Isolated horizontal gaze  evoked nystagmus | R | N | HR | R | N |
|  |  | Isolated vertical gaze  evoked nystagmus | R | N | R | R | N |
|  |  | Horizontal AND vertical  gaze evoked nystagmus | R | N | R | R | N |
|  |  | Rebound nystagmus | R | N | N | UN | N |
|  | **Gaze palsy (motility)** | Horizontal gaze palsy  (restricted motility) | R | R | HR | R | R |
|  |  | Vertical gaze palsy  (restricted motility) | R | HR | R | R | HR |
|  | **Nystagmus** | Downbeat nystagmus | R | N | R | R | N |
|  |  | Upbeat nystagmus | R | N | R | R | N |
|  |  | Horizontal spontaneous nystagmus | R | N | R | R | N |
|  |  | Central positional/-ing nystagmus | R | N | N | UN | N |
|  |  | Head-shaking nystagmus | R | N | N | N | N |
|  |  | Convergence retraction nystagmus | R | N | R | N | N |
|  |  | Periodic alternating nystagmus | R | N | N | R | N |
|  |  | Acquired pendular nystagmus | R | N | N | N | N |
|  | **Others** | Anisokoria | R | N | R | N | N |
|  |  | Ptosis | R | N | R | N | N |
