# Supplementary material for: An algorithm as a diagnostic tool for central ocular motor disorders, also to diagnose rare disorders
Source: Orphanet J Rare Dis. 2019 Aug 8;14:193. doi: 10.1186/s13023-019-1164-8 (PMC6688379; doi:10.1186/s13023-019-1164-8)
Supplement: Supplementary file 2 — Workflow of the algorithm – brain zones. These tables show the working principle of the algorithm using the example of 4 of the 8 brain zones (midbrain, pons, medulla, flocculus/paraflocculus). Whenever a symptom occurs in a patient, whether the brain zone’s score is increased depends on the type of linking: N = + 0, R = + 1, HR = + 2. (DOCX 20 kb) [file 13023_2019_1164_MOESM2_ESM.docx]

| **Additional File 2.1.**   \|  \| \| --- \| \| | | | | | **Midbrain** | | **Pons** | | **Medulla oblongata** | | **Flocculus/ paraflocculus** | |
| --- | --- | --- | --- | --- | --- | --- | --- | --- | --- | --- | --- | --- | --- |
|  |  |  |  |  |  |  |  |  |  |  |  |  |
| **Ocular  motor signs** | **Saccades** | | Impaired saccade initiation with  increased latency of saccades  ("ocular motor apraxia") | | N | | N | | N | | N | |
|  |  |  | Internuclear opthalmoplegia (INO),  aged < 60 years | | R | | R | | N | | N | |
|  |  |  | Internuclear opthalmoplegia (INO),  aged >= 60 years | | R | | R | | N | | N | |
|  |  |  | Horizontal saccade palsy | | N | | HR | | N | | N | |
|  |  |  | Vertical saccade palsy | | HR | | N | | N | | N | |
|  |  |  | NO Vertical saccade palsy | | N | | N | | N | | N | |
|  |  |  | Hypermetric saccades | | N | | N | | R | | N | |
|  | **Smooth  pursuit** | | Vertical saccadic smooth pursuit | | R | | N | | N | | R | |
|  |  |  | Horizontal saccadic smooth pursuit | | N | | R | | R | | R | |
|  |  |  | Impaired visual suppression  of the vestibulo-ocular reflex (VOR) | | N | | N | | N | | HR | |
|  | **Vestibular  signs** | | Pathological Head Impulse  Test (VOR-test) | | N | | N | | N | | R | |
|  |  |  | Skew deviation | | R | | R | | R | | N | |
|  |  |  | Head tilt | | R | | R | | R | | N | |
|  | **OKN** | | Horizontally reduced  optokinetic nystagmus | | N | | R | | N | | N | |
|  |  |  | Vertically reduced  optokinetic nystagmus | | R | | N | | N | | N | |
| **Additional File 2.2.**   \|  \| \| --- \| \| | | | | | | **Midbrain** | | **Pons** | | **Medulla oblongata** | | **Flocculus/ paraflocculus** |
|  |  |  |  |  |  |  |  |  |  |  |  |  |
| **Ocular  motor signs** | | **Gaze  holding** | | Isolated horizontal gaze  evoked nystagmus | | N | | HR | | R | | N |
|  |  |  |  | Isolated vertical gaze  evoked nystagmus | | HR | | N | | N | | N |
|  |  |  |  | Horizontal AND vertical  gaze evoked nystagmus | | R | | R | | R | | HR |
|  |  |  |  | Rebound nystagmus | | N | | R | | R | | HR |
|  |  | **Gaze palsy (motility)** | | Horizontal gaze palsy  (restricted motility) | | N | | HR | | R | | N |
|  |  |  |  | Vertical gaze palsy  (restricted motility) | | HR | | N | | N | | N |
|  |  | **Nystagmus** | | Downbeat nystagmus | | N | | R | | R | | HR |
|  |  |  |  | Upbeat nystagmus | | R | | R | | R | | N |
|  |  |  |  | Horizontal spontaneous nystagmus | | N | | R | | R | | N |
|  |  |  |  | Central positional/-ing nystagmus | | N | | N | | R | | N |
|  |  |  |  | Head-shaking nystagmus | | N | | N | | R | | N |
|  |  |  |  | Convergence retraction nystagmus | | HR | | N | | N | | N |
|  |  |  |  | Periodic alternating nystagmus | | N | | N | | N | | N |
|  |  |  |  | Acquired pendular nystagmus | | R | | R | | R | | N |
|  |  | **Others** | | Anisokoria | | R | | R | | R | | N |
|  |  |  |  | Ptosis | | R | | R | | R | | N |
