# Supplementary material for: An algorithm as a diagnostic tool for central ocular motor disorders, also to diagnose rare disorders
Source: Orphanet J Rare Dis. 2019 Aug 8;14:193. doi: 10.1186/s13023-019-1164-8 (PMC6688379; doi:10.1186/s13023-019-1164-8)
Supplement: Supplementary file 3 — Data entry mask with 3 examples. These tables show three sample patients’ signs and symptoms entered in the algorithm’s entry mask. “Yes” means the symptom was present, “No” means it was not and “0″ it was not looked for in the examination. The real diagnoses are:1 = PSP, 2 = Wernicke’s Encephalopathy, 3 = NPC. (DOCX 24 kb) [file 13023_2019_1164_MOESM3_ESM.docx]

**Additional File 3.1.**

| \|  \| \| --- \| \| | | | **Patient 1** | **Patient 2** | **Patient 3** |
| --- | --- | --- | --- | --- | --- | --- |
|  |  |  |  |  |  |
| **Age of onset** | | Age of onset: <10 years | No | No | Yes |
|  |  | Age of onset: ≥10 years | Yes | Yes | No |
| ***Rate of progression*** | | Years to decades | Yes | No | Yes |
|  |  | Months | No | No | No |
|  |  | Weeks | No | No | No |
|  |  | Days | No | Yes | No |
|  |  | Minutes to hours | No | No | No |
| ***Neurological symptoms*** | **Extrapyramidal symptoms** | Rigidity | Yes | No | No |
|  |  | Spasticity | No | No | No |
|  |  | Akinesia | Yes | No | Yes |
|  |  | Dystonia | No | No | Yes |
|  |  | Choreatoform | No | No | No |
|  |  | Resting tremor | No | No | 0 |
|  |  | Intention tremor | No | No | 0 |
|  | **Other  neurological symptoms** | Dysarthrophonia/dysphagia | Yes | Yes | Yes |
|  |  | Aphasia | No | No | No |
|  |  | Paresis (arms/legs/face) | No | No | No |
|  |  | Peripheral neuropathy | No | No | No |
|  |  | Ataxia | No | Yes | Yes |
|  |  | Seizures | No | Yes | Yes |
|  |  | Disoriented to time, place,  person or situation | No | Yes | 0 |
| ***Psychiatric symptoms*** | **Childhood** | Mental retardation  (mild to moderate: IQ 35-69) | No | No | Yes |
|  |  | Mental retardation  (severe to profound: IQ up to 34) | No | No | No |
|  |  | Developmental disorder of  speech and language | No | No | 0 |
|  |  | Developmental disorder  of scholastic skills | No | No | 0 |
|  |  | Hyperkinetic disorder | No | No | 0 |
|  |  | Conduct disorder  (beginning in childhood) | No | No | No |
|  | **Adulthood** | Amnesic disorder | No | Yes | 0 |
|  |  | Mood disorder | Yes | No | 0 |
|  |  | Psychotic disorder | No | No | No |
|  |  | Conduct (behavioral) disorder | No | No | No |
| **Other Symptoms** | | Fever | No | No | 0 |

| **Additional File 3.2.**   \|  \| \| --- \| \| | | | **Patient 1** | **Patient 2** | **Patient 3** |
| --- | --- | --- | --- | --- | --- | --- |
|  |  |  |  |  |  |
| **Ocular motor signs** | **Saccades** | Impaired saccade initiation with  increased latency of saccades  ("ocular motor apraxia") | No | 0 | No |
|  |  | Internuclear opthalmoplegia (INO),  aged < 60 years | No | No | No |
|  |  | Internuclear opthalmoplegia (INO),  aged >= 60 years | No | No | No |
|  |  | Horizontal saccade palsy | Yes | 0 | 0 |
|  |  | Vertical saccade palsy | Yes | 0 | Yes |
|  |  | NO Vertical saccade palsy | No | 0 | No |
|  |  | Hypermetric saccades | No | 0 | No |
|  | **Smooth pursuit** | Vertical saccadic smooth pursuit | No | Yes | Yes |
|  |  | Horizontal saccadic smooth pursuit | Yes | Yes | 0 |
|  |  | Impaired visual suppression of  the vestibulo-ocular reflex (VOR) | 0 | 0 | 0 |
|  | **Vestibular signs** | Pathological Head Impulse Test  (VOR-test) | 0 | 0 | 0 |
|  |  | Skew deviation | No | No | No |
|  |  | Head tilt | No | No | No |
|  | **OKN** | Horizontally reduced  optokinetic nystagmus | 0 | 0 | 0 |
|  |  | Vertically reduced  optokinetic nystagmus | 0 | 0 | Yes |

| **Additional File 3.3.**   \|  \| \| --- \| \| | | | **Patient 1** | **Patient 2** | **Patient 3** |
| --- | --- | --- | --- | --- | --- | --- |
|  |  |  |  |  |  |
| **Ocular motor signs** | **Gaze holding** | Isolated horizontal gaze  evoked nystagmus | No | Yes | No |
|  |  | Isolated vertical gaze  evoked nystagmus | No | No | No |
|  |  | Horizontal AND vertical gaze  evoked nystagmus | No | No | No |
|  |  | Rebound nystagmus | 0 | 0 | 0 |
|  | **Gaze palsy (motility)** | Horizontal gaze palsy  (restricted motility) | Yes | 0 | 0 |
|  |  | Vertical gaze palsy  (restricted motility) | Yes | 0 | 0 |
|  | **Nystagmus** | Downbeat nystagmus | No | No | No |
|  |  | Upbeat nystagmus | No | Yes | No |
|  |  | Horizontal spontaneous nystagmus | No | No | No |
|  |  | Central positional/-ing nystagmus | 0 | 0 | 0 |
|  |  | Head-shaking nystagmus | 0 | 0 | 0 |
|  |  | Convergence retraction nystagmus | No | No | No |
|  |  | Periodic alternating nystagmus | No | No | No |
|  |  | Acquired pendular nystagmus | No | No | No |
|  | **Other** | Anisokoria | No | No | No |
|  |  | Ptosis | No | No | No |
