# Supplementary material for: An algorithm as a diagnostic tool for central ocular motor disorders, also to diagnose rare disorders
Source: Orphanet J Rare Dis. 2019 Aug 8;14:193. doi: 10.1186/s13023-019-1164-8 (PMC6688379; doi:10.1186/s13023-019-1164-8)
Supplement: Supplementary file 4 — Output of the algorithm for the input of the 3 examples from Additional file 3. According to our result interpretation rules (see Methods), the algorithm’s diagnosis suggestions for the three patients are: Patient 1: brain zone: Basal ganglia and pons; disease: PSP. Patient 2: brain zone: Pons, medulla oblongata, flocculus/paraflocculus and vermis/fastigial nucleus; disease: Wernicke’s encephalopathy, MS and inflammatory encephalitis. Patient 3: brain zone: Midbrain, basal ganglia; disease: NPC. The real diagnoses are: 1 = PSP, 2 = Wernicke’s Encephalopathy, 3 = NPC. (DOCX 16 kb) [file 13023_2019_1164_MOESM4_ESM.docx]

**Additional File 4.**

|  | **Patient 1** | **Patient 2** | **Patient 3** |
| --- | --- | --- | --- |
|  |  |  |  |
| **Zones** |  |  |  |
| **Midbrain** | 4 | 2 | 4 |
| **Pons** | 5 | 4 | 0 |
| **Medulla oblongata** | 2 | 3 | 0 |
| **Flocculus/paraflocculus** | 1 | 3 | 2 |
| **Vermis/fastigial nucleus** | 1 | 3 | 2 |
| **Nodulus/uvula** | 0 | 1 | 1 |
| **Basal ganglia** | 6 | 0 | 3 |
| **Fronto-parietal cortex** | 0 | 0 | 0 |
|  |  |  |  |
| **Illness** |  |  |  |
| **Cerebellar syndromes** | 5 | 7 | 8 |
| **Inflammatory encephalitis** | 8 | 10 | 7 |
| **Tumor** | 8 | 9 | 7 |
| **Infarction/ hemorrhage** | 4 | 6 | 2 |
| **Multiple sclerosis** | 8 | 10 | 6 |
| **Parkinsonian syndromes** | 10 | 4 | 4 |
| **Progressive supranuclear palsy (PSP)** | 14 | 1 | 6 |
| **Wernicke’s encephalopathy** | 3 | 12 | 1 |
| **Ataxia tele-angiectasia** | 5 | 4 | 9 |
| **Ataxia with oculomotor apraxia type 1 and 2 (AOA1/2)** | 7 | 6 | 9 |
| **Gaucher’s disease Type 3 (GD3)** | 10 | 5 | 9 |
| **Huntington’s chorea (HTT)** | 4 | 6 | 9 |
| **Niemann-Pick disease Type C (NP-C)** | 11 | 5 | 12 |
| **Tay-Sachs disease** | 9 | 5 | 9 |
